# Comparison and validation of two mathematical models for the impact of mass drug administration on *Ascaris lumbricoides* and hookworm infection

**DOI:** 10.1016/j.epidem.2017.02.001

**Published:** 2017-03

**Authors:** Luc E. Coffeng, James E. Truscott, Sam H. Farrell, Hugo C. Turner, Rajiv Sarkar, Gagandeep Kang, Sake J. de Vlas, Roy M. Anderson

**Affiliations:** a Department of Public Health, Erasmus MC, University Medical Center Rotterdam, Rotterdam, The Netherlands; b London Centre for Neglected Tropical Disease Research, Department of Infectious Disease Epidemiology, St. Mary’s Campus, Imperial College London, London WC2 1 PG, United Kingdom; c Division of Gastrointestinal Sciences, Christian Medical College, Vellore 632004, Tamil Nadu, India

**Keywords:** MDA, mass drug administration, PCT, preventive chemotherapy, preSAC, pre-school age children (age 2–5), SAC, school age children (age 5–15), SMC, sequential Monte Carlo, STH, soil-transmitted helminths, WHO, World Health Organization, Soil-transmitted helminths, Mass drug administration, Mathematical modelling, Validation, Model comparison

## Abstract

The predictions of two mathematical models of the transmission dynamics of *Ascaris lumbricoides* and hookworm infection and the impact of mass drug administration (MDA) are compared, using data from India. One model has an age structured partial differential equation (PDE) deterministic framework for the distribution of parasite numbers per host and sexual mating. The second model is an individual-based stochastic model. Baseline data acquired prior to treatment are used to estimate key transmission parameters, and forward projections are made, given the known MDA population coverage. Predictions are compared with observed post-treatment epidemiological patterns. The two models could equally well predict the short-term impact of deworming on *A. lumbricoides* and hookworm infection levels, despite being fitted to different subsets and/or summary statistics of the data. As such, the outcomes give confidence in their use as aids to policy formulation for the use of PCT to control *A. lumbricoides* and hookworm infection. The models further largely agree in a qualitative sense on the added benefit of semi-annual *vs.* annual deworming and targeting of the entire population *vs.* only children, as well as the potential for interruption of transmission. Further, this study also illustrates that long-term predictions are sensitive to modelling assumptions about which age groups contribute most to transmission, which depends on human demography and age-patterns in exposure and contribution to the environmental reservoir of infection, the latter being notoriously difficult to empirically quantify.

## 1 Introduction

Globally, over 1 billion people are infected with soil-transmitted helminths (STH) (Hotez et al., 2014). The majority of infecting STH species are *Ascaris lumbricoides* (roundworm), *Trichuris trichiura* (whipworm), and hookworm (*Necator americanus* and *Ancyclostoma duodenale*). Because morbidity due to STH is highly correlated with intensity of infection, and roundworm and whipworm infection levels are highest in children, the current main control approach consists of preventive chemotherapy (PCT) targeted at school age children (SAC) and pre-school age children (preSAC) (World Health Organization, 2012a, World Health Organization, 2012b). This approach is highly effective at reducing the infection burden of roundworm and whipworm in children, but has limited impact on the burden of hookworm in adults, in whom hookworm infection levels are typically highest (Anderson et al., 2013, Coffeng et al., 2015, Truscott et al., 2015). In contrast, hookworm is more effectively controlled by means of PCT targeting the entire population, also referred to as mass drug administration (MDA). Although MDA is not the main STH control strategy, it is already effectively taking place in areas co-endemic for the helminth infections lymphatic filariasis and onchocerciasis that are being controlled with MDA using drugs that also impact STH. Typically, shortly after administration of targeted PCT or MDA, STH infection levels in the population bounce back to their pre-perturbation equilibrium state in a predictable manner. This is the result of the presence of a reservoir of infection in the environment (soil contaminated with faecal matter containing worm eggs or larvae) and is further exacerbated by density-dependent processes operating on worm population growth (Anderson and May, 1985). Because there is no strong immune response in STH infections, after deworming, individuals reacquire new infections at rates that depend on exposure to the environmental reservoir (i.e. ingestion of contaminated soil for ascariasis and trichuriasis, and exposure to free-living hookworm larvae that may burrow through exposed skin). Given the relatively short lifespan of STH of one to three years, bounce back can be rapid, and, in the case of *A. lumbricoides* in high transmission settings, even within one year (Anderson and May, 1985, Seo et al., 1983, Thein-Hlaing et al., 1990).

Mathematical models play an important role in understanding the transmission of STH and the impact of control measures (Anderson and May, 1991, Anderson and May, 1985, Truscott et al., 2016). To have confidence in the ability of mathematical models to inform policy makers, and in particular the feasibility of the ambitious goal of achieving <1% prevalence of moderate and heavy intensity infection by 2020 (World Health Organization, 2012b), it is important to validate model predictions against appropriate epidemiological data. Ideally, model validation is based on data from field studies that include at least one round of PCT and surveys of the intensity of infection in different age classes (fully cross sectional by age and gender), both before and after treatment. Here, we present such a validation of two previously developed transmission models for STH (Coffeng et al., 2015, Truscott et al., 2015). The Bill and Melinda Gates Foundation (BMGF) recently funded a consortium of research groups to develop and exploit mathematical models of the transmission dynamics and impact of control measures of nine neglected tropical diseases (NTDs) (http://www.ntdmodelling.org). Two or three groups were funded to address each of the chosen infectious diseases to allow comparisons of the predictions of different mathematical models relating to how various control measures impact the prevalence and intensity of infection in defined settings (Hollingsworth et al., 2015). We investigate whether the two STH models are accurate in their predictions of the impact of defined mass drug administration control programmes, given information on the pre-control epidemiological situation and drug treatment coverage. The results of validation and the comparison of the results from the two models helps to: (1) illuminate the role of the different population processes, (2) focus on whether the assumptions incorporated within the models are sufficient to mimic observed patterns, and (3) point to what additional data is of value to improve the accuracy of model predictions and hence refine policy formulation for the control of STH infections by mass drug administration.

## 2 Methods

In this paper we compare two transmission models for STH (Table 1) developed independently by research groups at Imperial College London (ICL) and Erasmus MC, University Medical Center Rotterdam (Erasmus MC). The ICL model is deterministic and takes the form of a set of partial differential equations that describe the dynamics of the mean worm burden in the host population as a function of time and host age. The mean worm burden describes the distribution of worms in individuals through a negative binomial distribution with a fixed aggregation parameter (Anderson et al., 2014, Anderson and May, 1985, Truscott et al., 2016, Truscott et al., 2015, Truscott et al., 2014). The Erasmus MC model is stochastic and individual-based, in terms of both hosts and intestinal parasite numbers per host, and deterministic with regards to the environmental reservoir of infection (Coffeng et al., 2015). Both models are based on similar biological and demographic assumptions (Anderson and May, 1991, Anderson and May, 1985), but differ in the manner and detail in which they are implemented. Both models can generate probability distributions for predicted worm burden and egg output for individuals of a particular age at a given time. The output can therefore be directly compared to observed epidemiological data, for the purposes of parameter estimation and model validation, as well as for model comparisons.

We assessed model performance by comparing model predictions to time series data on *A. lumbricoides* and hookworm infection levels in populations during PCT. For this purpose, we first fitted both models to detailed pre-control epidemiological data on age-specific infection levels of *A. lumbricoides* (Elkins et al., 1988, Elkins et al., 1986) and hookworm from India (Sarkar et al., 2017). Next, we projected forward over time to predict post-control patterns of infection, given information on the timing and population coverage of PCT. Model calibration was conducted separately by the two groups, based on common data sources or published literature (Albonico et al., 2003, Anderson et al., 2014, Anderson and May, 1985, Anderson and May, 1982, Anderson and Schad, 1985, Augustine, 1923, Bethony et al., 2006, Brooker et al., 2004, Coffeng et al., 2015, Croll et al., 1982, Elkins et al., 1986, Hotez et al., 2004, Jambulingam et al., 2016, Levecke et al., 2014, Pullan et al., 2010, Truscott et al., 2016, Truscott et al., 2014, Vercruysse et al., 2011) (see Supplemental Table S1 for details), but using different approaches (more details below). Last, the two sets of model predictions were compared to see how well they could reproduce the post-control infection intensity and prevalence data.

### 2.1 Model description

#### 2.1.1 ICL model

The model developed by ICL is a deterministic description of parasite natural history, transmission and PCT impact. See prior publications for a detailed exposition (Anderson et al., 2014, Truscott et al., 2016, Truscott et al., 2015, Truscott et al., 2014). In the deterministic model used here, the distribution of worm burdens among individuals of a given age and at a given time is assumed to be negative binomial with a fixed aggregation parameter, *k_w_*. Partial differential equations describe the evolution over time and age of the mean host worm burden in a fully age-structured host population and also the dynamics of a single shared environmental reservoir of larval infective stages. It assumes that the parasite is dioecious and polygamous and has density-dependent egg production. In the model, it is assumed that the age-intensity profiles are generated by age-dependent exposure to the infective larvae in the environment and not by acquired immunity. The model implicitly includes certain sources of variability, by averaging over all possible combinations of male and female worm burdens for a given mean value. Worm burdens can be used to calculate the probability of a given egg output for an individual at any time and also to calculate the likelihood of a given observed dataset recording egg output and worm burdens for individuals in each age grouping. Though the model has full age structure, the outputs can be grouped into the treatment age groupings such as SAC (5 to14 years of age) and adults (≥15 years of age), which are used by countries and WHO to track the impact of control interventions.

#### 2.1.2 Erasmus MC model

The Erasmus MC model (called WORMSIM, version 2.58Ap9) is a generalised individual-based modelling framework for transmission and control of helminth infections in humans (Coffeng et al., 2015), and has been previously used to predict the impact of control interventions against onchocerciasis (Stolk et al., 2015), lymphatic filariasis (Jambulingam et al., 2016), and hookworm infection (Coffeng et al., 2015). WORMSIM simulates the life histories of a discrete number of individual humans and individual worms within those humans, which are born and die in a stochastic fashion. Simulated humans are exposed and contribute to a central reservoir of infection in the external environment, in which infective material (e.g. worm larvae or eggs) survive in an exponential fashion (at each time step in the simulation, a fixed proportion of the reservoir decays). Infective material is produced by female worms after a period of pre-patency (maturation in the human host), and only when at least one male worm is present in the same host. The degree of parasite aggregation within the human population is governed by the level of inter-individual variation in exposure to the central reservoir of infection (by age, sex, and random individual factors). Similarly, the model allows for heterogeneity in participation in PCT, as well as systematic non-compliance to PCT. The model further accounts for different sources of variation, such as measurement error in parasitological test outcomes (any arbitrary parasitological test based on egg counts can be simulated, e.g. Kato-Katz faecal smear (World Health Organization, 1994)). Model code and installation and user instructions have been published elsewhere (Coffeng et al., 2015) or can be obtained on request.

### 2.2 Epidemiological data

#### 2.2.1 Ascariasis

The data used to validate the ascariasis models constitute worm expulsion data from the mass drug administration arm (two villages) of a field trial in Pulicat, India, previously described elsewhere (Elkins et al., 1988, 1986). In short, at baseline (January 1984) the study population of age 1–70 was dewormed using pyrantel pamoate (68% overall population coverage) and treated subjects were asked to collect stools over the next 48 h to count the number of expulsed worms. In November 1984, another round of MDA with pyrantel pamoate took place and again stools were collected and expelled worms were counted.

#### 2.2.2 Hookworm

The hookworm data are from a recently concluded cluster randomised community-intervention trial conducted in a tribal population in Tamil Nadu, India that has the aim to assess the effectiveness of MDA in reducing the level of hookworm infection (Sarkar et al., 2017). In the current modelling exercise we selected data from the three most highly endemic villages (out of 45 villages), since these had the highest number of infected individuals before and after treatment and hence offered the most information to validate the models against. All three villages received four rounds of treatment – two rounds at the beginning (months zero and one), followed by another two rounds after six months (months seven and eight). Three faecal samples were collected from each participant during the baseline and follow-up surveys. Follow-up surveys were planned at three-monthly intervals after the second (at 4 and 7 months) and the last MDA rounds (11, 14, 17, 20 months, see Supplemental Fig. S1 for an overview of the study timeline, see elsewhere (Sarkar et al., 2017) for details). Although single faecal samples were also collected shortly after each MDA round (at 0, 1, 7 and 8 months), we only used the data based on triple samples as the sensitivity of the single egg counts was considered to low to usefully assess model performance. All faecal samples were tested for the presence of hookworm ova using the formol-ether technique (Allen and Ridley, 1970). Positive samples were retested by the McMaster egg counting technique (Levecke et al., 2011) to quantify the number of eggs per gram (epg) of faeces. Infection intensity in individuals was categorized into light, moderate, or heavy infection (as previously defined (Albonico et al., 2003), see also Supplemental Table S1), based on the average of the three egg counts at a given point in time.

Given the success of community-wide treatment in this study, many of the follow-up time-points had too few detected hookworm infections to enable a meaningful comparison with model output (i.e. many egg counts of zero). For this reason, we chose to base the quantitative comparison on data from four and seven months after the start of the treatment programme, the last of which immediately precedes the third MDA round. For validation of the ICL model, data from one of the three villages was used (45 individuals for the pre-control survey); to validate the Erasmus MC model, data from the three villages (N = 135 for the pre-control survey) were aggregated to allow for useful statistical inference (data from a single village constituted too few individuals to allow meaningful estimation of transmission parameters, given that the model also incorporates stochastic uncertainty in its predictions).

### 2.3 Parameter estimation

#### 2.3.1 ICL model

The deterministic nature of the ICL model makes constructing a likelihood and investigating its distribution relatively straightforward (Truscott et al., 2016). The model was fitted to individual-level baseline data on infection levels stratified by age (i.e. before deworming). Because the data on hookworm infection levels constituted egg counts, first a model of egg output distribution was parameterised. There is good evidence that the distribution of egg counts under repeated measurements generated by an infected individual with a constant number of parasites is negative binomial in form, as previously observed for hookworm (Anderson and Schad, 1985). This appears to be true across a range of helminth species. The availability of three individual samples of hookworm egg counts for each individual allowed an estimate of the aggregation parameter associated with the negative binomial distribution for eggs in a given sample, *k_e_* (Truscott et al., 2016). In a separate stage of fitting, a likelihood function for the age-stratified mean epg (hookworm) and worm burden data (ascariasis) was constructed. This likelihood was a function of parameters for the age-dependent exposure and contribution to the environmental reservoir, overall transmission intensity, worm aggregation with hosts, density-dependent fecundity and the net egg output per female worm (Truscott et al., 2016). Information from the data was insufficient to specify all parameters and so additional prior distributions were used, based on ranges derived from the literature (Anderson and May, 1991) (see Supplemental Table S1 for details).

#### 2.3.2 Erasmus MC model

The Erasmus MC model was fitted to reproduce pre-control infection levels by age group in terms of either average number of adult female worms (ascariasis) or the distribution of no, low, moderate, and heavy infection based on McMaster egg counts (hookworm). For hookworm, we first estimated the variation in repeated McMaster egg counts using a separate statistical model (as done for the ICL model), assuming that repeated egg counts from individuals follow a negative binomial distribution with aggregation parameter *k_e_* (Supplemental Table S1). Conditional on the estimate of *k_e_* and our previously used assumption that host contribution and exposure to the environmental reservoir for hookworm infection increase linearly with age until age 10 and are stable from then onwards (Coffeng et al., 2015), we estimated parameter values for the overall transmission rate and random inter-individual variation in exposure and contribution to the environmental reservoir of infection (the latter being equivalent to the aggregation parameter *k_w_* for parasite aggregation in the host population in the ICL model). Because the data for ascariasis constituted worm counts, we could use a previous estimate of *k_w_* = 0.8 (Elkins et al., 1986) and did not need to quantify *k_e_* for variation in egg counts.

Parameter values for both worm species were estimated using a two-step approach. First we explored the parameter space by means of a grid search to identify where most of the posterior probability mass was situated, using the average of 50 repeated simulations as the model expectation (effectively removing most model stochasticity). Second, in a Bayesian framework we refined our parameter estimates and their uncertainty by accounting for stochasticity using sequential Monte Carlo (SMC) (Filippi et al., 2011, Toni et al., 2009). To allow stochastic effects to be as realistic as possible, a population of about 600 individuals was simulated, which is similar to the size of the census population in the three villages (N = 577). Log-normal priors for the transmission rate and level of inter-individual variation were centred at the point estimates from the grid search, but were defined to be only weakly informative. Using SMC, we produced 250 samples from the approximate joint posterior distribution of parameter values for the transmission rate and inter-individual variation in exposure. To check the quality of the sample from the approximate joint posterior distribution, we calculated the effective sample size of posterior samples (a measure of the level of Monte Carlo error) based on the weights of the posterior samples (Cornebise et al., 2008).

## 3 Results

### 3.1 Ascariasis

**Fig. 1:**
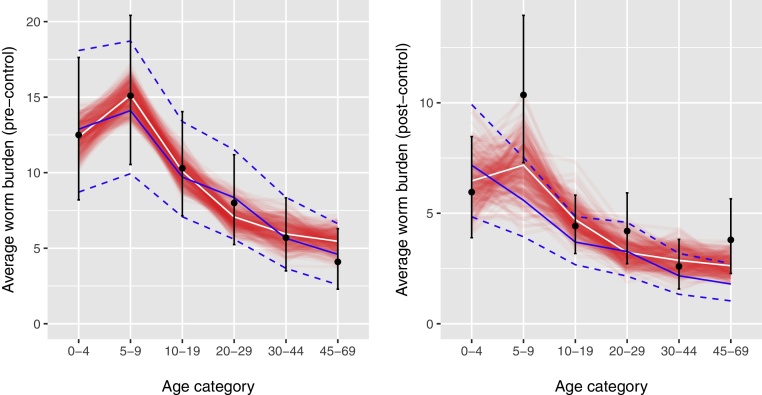
Comparison of model predictions for Ascaris lumbricoides infection to data. The deterministic ICL model (blue) and the stochastic individual-based Erasmus MC model (white) were calibrated to reproduce data on age patterns in pre-control infection levels (black bullets with 95% confidence intervals, left panel) in Pulicat, India (Elkins et al., 1988, Elkins et al., 1986). Next, forward predictions were made given information on the timing and coverage of mass drug administration. The right panel compares the reported worm expulsion data and the predicted age profile in infection levels at 11 months after a single round of mass drug administration with pyrantel pamoate targeting all individuals between age 1 and 70, implemented at 68% coverage of the overall population, assuming that the drug kills 95% of the worms in treated individuals (Elkins et al., 1988, Elkins et al., 1986). Predictive uncertainty (i.e. model parameter uncertainty) for the ICL model is represented by the dashed blue lines, which represent 95%-Bayesian credible intervals; for the Erasmus MC model, predictive uncertainty (i.e. stochastic and model parameter uncertainty) is represented by a set of 250 stochastic simulations (red lines) that approximate the predictive posterior distribution (see Methods section for details).

Fig. 1 illustrates the comparison of predictions by the ICL and Erasmus MC models for *A. lumbricoides* infection to data. Both models were fitted to pre-control age patterns in average adult female worm counts (i.e. adult female worms expulsed after a first treatment with pyrantel pamoate) and could very well reproduce these age patterns (left panel). Transmission parameter estimated based on the pre-control data showed considerable correlation, both for the Erasmus MC model (Supplemental Fig. S2) and the ICL model (Supplemental Fig. S3), highlighting the appropriateness of using Bayesian methods to account for multiple possible parameter combinations. Then both models were run forward in time assuming that 68% of the overall population were treated with pyrantel pamoate (as reported for the field study), and model predictions for the average adult female worm burden at the time point 11 months after the first MDA round were compared to the observed number of adult female worms expulsed after a second treatment with pyrantel pamoate (right panel). Both models could adequately reproduce the overall post-control worm density, given the high level of uncertainty in the data. Still, the reported peak worm load in children aged five to nine was higher than either model predicted, where the Erasmus MC model tended to fit this peak better than ICL model. The latter can be explained by the fact that for the ICL model, age-dependent exposure to the environmental reservoir (relative to the reference value of 1.0 for children of age 10–19) was estimated to start at 1.6 for ages 0–4 years and then to decline to 1.54 for ages 5–9 (Supplemental Fig. S4), whereas in the Erasmus MC model age-dependent exposure (relative to the exposure at age of highest exposure) was estimated to increase from 0.33 at age zero to 1.0 at age 2.9 and only then decrease again (see Supplemental Table S1 for more details). These different estimates for the age patterns arise from different estimation approaches (assuming piece-wise linear vs. piece-constant relative exposure) and the different types of data that they were estimated from (ICL: individual egg counts; Erasmus MC: prevalences of none, light, moderate, and heavy infection). Further, in general, the ICL model predicted somewhat lower infection levels for the post-control situation than the Erasmus MC model.

**Fig. 2:**
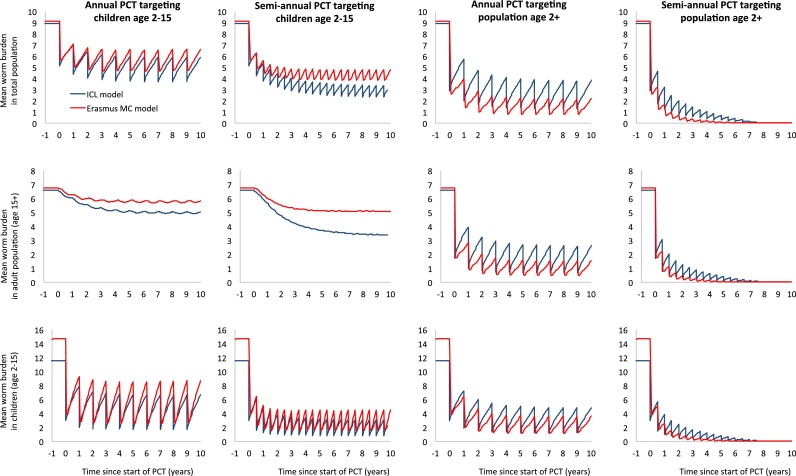
Comparison of model predictions for the impact of different preventive chemotherapy strategies on the A. lumbricoides worm burden in different age groups. Both models were calibrated to predict a pre-control average female worm load of 9 worms per person in the general population, conditional on the assumption that the level of parasite aggregation is k_w_ = 0.8. Further, in both models seventy-five percent of the PCT target population were assumed to be treated with albendazole, which was assumed to kill 99% of the worms in treated people. The parameters for worm biology and age patterns in exposure and contribution to the reservoir are as described in Supplemental Table S1 and differ between the two models.

In Fig. 2 we further compare the two models, but now for long-term predictions over a ten-year period for the impact of various deworming strategies at 75% coverage of the target population (i.e. the operational target set by WHO) on *A. lumbricoides* infection levels. Both models were calibrated to reproduce the same average worm burden in the general population, but with independent and sometimes different assumptions about worm biology and transmission patterns as also used for the validation described above (Supplemental Table S1). Model predictions for the mean worm burden during annual PCT largely agree, although the Erasmus MC model predicts somewhat higher infection levels in the children (both pre and post-control), suggesting that this age group contributes more to transmission in the Erasmus MC model. In both models, an age group’s contribution to transmission is driven by assumptions about human demography (Indian demography for Erasmus MC and Ugandan demography for the ICL model – an assumption to which the ICL predictions were robust in sensitivity analyses), age-dependent contribution to the reservoir (proportional to exposure in the ICL model, and rising with age in the Erasmus MC model), and age-dependent exposure to the reservoir (similar in both models, although the age of peak exposure is somewhat higher in the Erasmus MC model, see Supplemental Fig. S4). Both models further agree that semi-annual deworming and targeting of the entire population (age two and above) lead to a significantly stronger reduction in infection levels, although Erasmus MC model predicts a larger added benefit of targeting the whole population than the ICL model. The models further agree that to interrupt transmission in this scenario, semi-annual deworming of the entire population is necessary, with the Erasmus MC model predicting earlier achievement of elimination than the ICL model.

### 3.2 Hookworm

Because the hookworm data have only been published recently (only trial design and baseline results (Sarkar et al., 2017)), we provide a short summary of the data. The total census population of the three highest endemic villages consisted of a total of 577 persons, of which 557 were two years or older and were therefore eligible for treatment and parasitological testing. The parasitological data consisted of 737 triple egg counts that were collected over time in 208 unique individuals. Of these triple egg counts, 135 were measured before the start of MDA and 107, 123, 109, 76, 87, and 100 at months 4, 7, 11, 14, 17, and 20, respectively. The crude pre-control prevalences of light, moderate and heavy infection were 33.3%, 1.5%, and 2.2% (total prevalence of infection 37.0%). Given the individual village sample sizes (45, 43, and 47 participants), prevalences of overall infection (35.5%, 44.2%, and 31.9%) and moderate-to-heavy intensity infection (6.7%, 2.3%, 2.1% or 3, 1, and 1 case) were very similar in the three villages. The average egg counts in the first two villages (544 and 314 epg) were higher than that of the third village (132 epg). The overall population coverage of the four MDA rounds was 61.5%, 62.6%, 67.4%, and 64.0% (i.e. the percentage of census population treated).

**Fig. 3:**
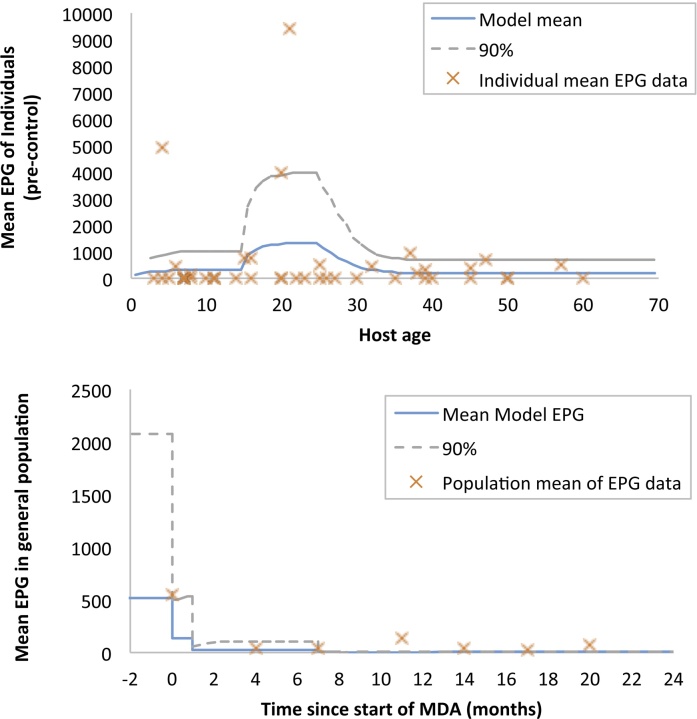
Comparison of ICL model predictions for hookworm infection to data. Transmission parameters of the ICL model were fitted to individual-level epg data (top panel). The solid line gives the mean epg predicted by the model using the maximum likelihood parameters values. Red crosses are the individual epg counts and the dashed line denotes the predictive interval within which 90% of the observations are predicted to fall by the model. Next, the model was allowed predict forward trends in infection, given information on coverage and timing of mass drug administration (bottom panel). The blue line gives the mean epg across all ages in the community while the grey line tracks the 90% predictive interval for epg readings across all ages.

**Fig. 4:**
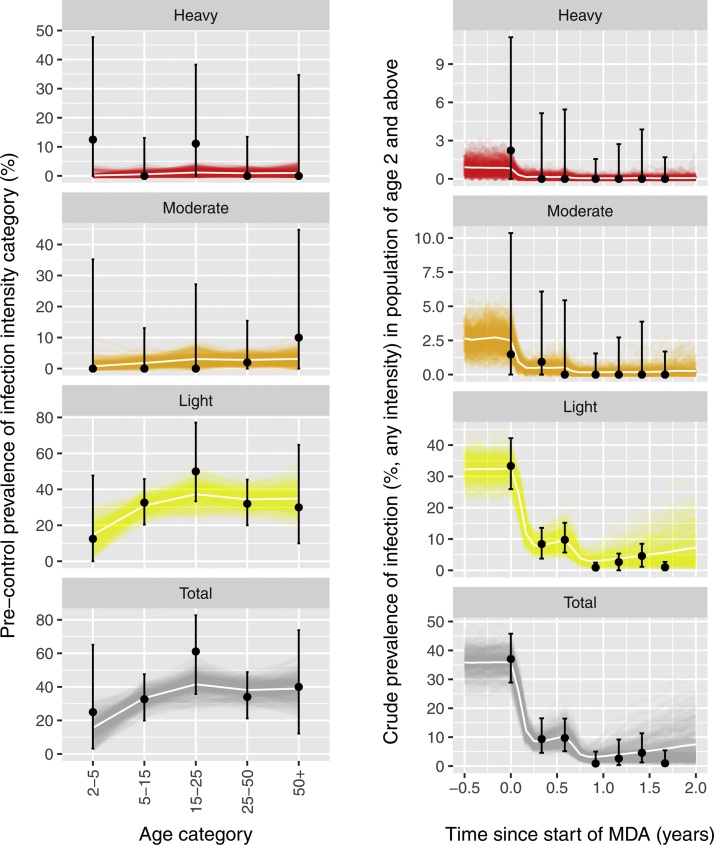
Comparison of Erasmus MC model predictions for hookworm infection to data. Transmission parameters of the Erasmus MC model were fitted to age-specific prevalence of light, moderate, and heavy infection (left column of panels). Predictive uncertainty (i.e. stochastic and model parameter uncertainty) is represented by a set of 250 stochastic simulations (lines) that approximate the predictive posterior distribution (see Methods section for details); the white line represents the posterior average. Next, the model was allowed predict forward trends in infection, given information on coverage and timing of mass drug administration (right column of panels).

**Fig. 5:**
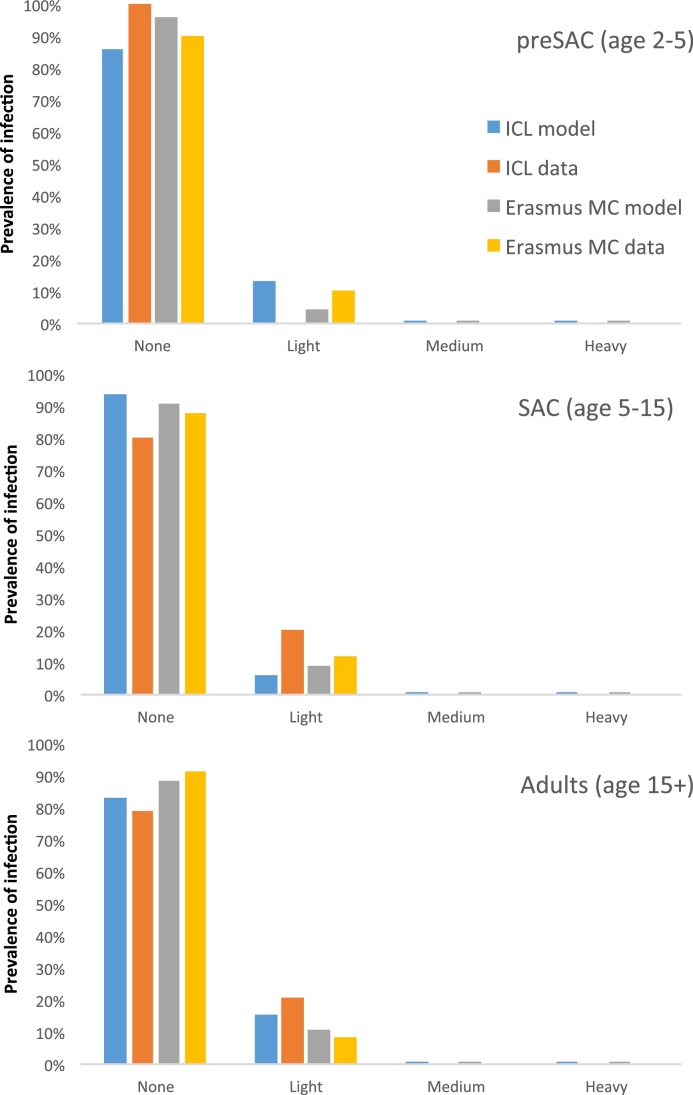
Comparison of predictive performance of the ICL and Erasmus MC models for hookworm infection at six months after the last of two monthly rounds of mass drug administration. The two models were compared to different but similar subsets of the trial data (blue: data from one village for ICL, N = 37; grey: aggregate data from three villages for Erasmus MC, N = 123). Model predictions (orange and yellow bars) were in good and comparable agreement with the data. Comparisons are made for three age groups: pre-school age children (preSAC), school age children (SAC), and adults.

Fig. 3, Fig. 4 illustrate the comparison of predictions by both models for hookworm infection to data. Each of the models was fitted to age-specific baseline data on infection levels. The ICL model was fitted to pre-control individual epg data (mean epg of three counts per person), while the Erasmus MC model was fitted to pre-control prevalence of infection intensity categories by age. Parameter estimates for the baseline fit are described in Supplemental Table S1. Again, transmission parameter estimated based on the pre-control data showed considerable correlation, both for the Erasmus MC model (Supplemental Fig. S5) and the ICL model (Supplemental Fig. S6), highlighting the appropriateness of using Bayesian methods. Next, both models were run forward in time and the impact of mass drug administration was predicted up to two years after the start of MDA with albendazole. Both models adequately reproduced both the pre-control and the first year of post-control data, given the high level of uncertainty in the data. In Fig. 5, we provide a more detailed comparison of the predictive performance of both models in terms of the distribution of intensity of infection at six months after the second MDA round (i.e. seven months after start of MDA), again illustrating good model agreement, even though the models were calibrated to different subsets of the data. For the last four data points in the time series (month 11, 14, 17, and 20), the data held only a few cases; for this period the ICL model predicted that transmission is probably interrupted, whereas the Erasmus MC model predicted that although infection levels were likely to stay low, there was the possibility of transmission picking up again. This difference is due to different estimates for the level of parasite aggregation (Supplemental Table S1), with the Erasmus MC model estimating higher overall parasite aggregation within age groups (but a smoother age pattern) than the ICL model; and differences in model structure: because of its deterministic nature, the ICL model will only predict sustained transmission or interruption of transmission, and will thus tip over to the one or the other, whereas the individual-based nature of the Erasmus MC model allows prediction of sustained *vs.* interrupted transmission in a probabilistic manner.

**Fig. 6:**
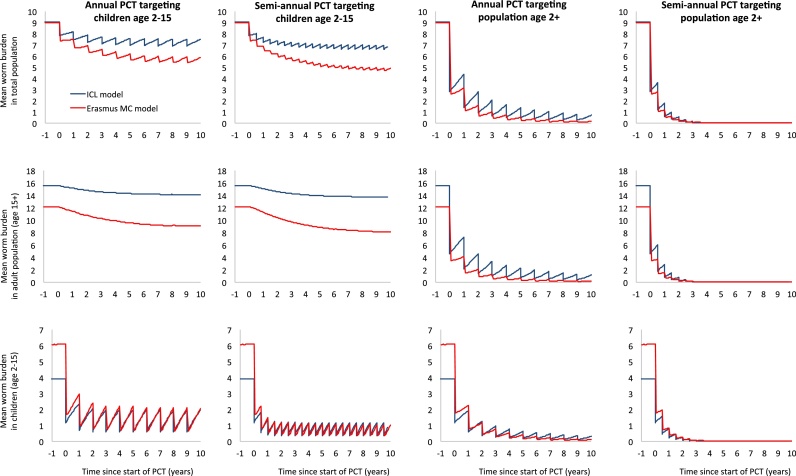
Comparison of model predictions for the impact of different preventive chemotherapy strategies on the hookworm burden in different age groups. Both models were calibrated to predict a pre-control average female worm load of 9 worms per person in the general population, conditional on the assumption that the level of parasite aggregation is assumed to be k_w_ = 0.35. Further, in both models seventy-five percent of the PCT target population we assumed to be treated with albendazole, which was assumed to kill 95% of the worms in treated people. The parameters for worm biology and age patterns in exposure and contribution to the reservoir are as described in Supplemental Table S1 and differ between the two models.

In Fig. 6 we further compare the two models, but now for long-term predictions over a ten-year period for the impact of various deworming strategies implemented at 75% overage of the target population (i.e. the operational target set by WHO) on hookworm infection levels. Both models were calibrated to reproduce the same average worm burden in the general population, but with independent and sometimes different assumptions about worm biology and transmission patters as also used for the validation described above (Supplemental Table S1). Due to differences in assumption about human demography and age-dependent exposure and contribution to the reservoir (which are assumed proportional to each other in both models), the Erasmus MC model attributes more of the transmission to children, such that pre-control infection levels in children are higher, but the net impact of deworming children at population level is higher. Again, the two models agree on the larger impact of semi-annual deworming and targeting of the entire population (age two and above). The models further agree on the potential of achieving elimination through targeting of the whole population, with predict.

## 4 Discussion

We present a comparison of predictions of two structurally similar models for transmission of soil-transmitted helminths that have different implementation (deterministic *vs.* individual-based) and different parameter values. The two models could equally well predict the short-term impact of deworming on *A. lumbricoides* and hookworm infection levels as reported in two sets of field trial data from India, despite being fitted to different subsets and/or summary statistics of the data. As such, the outcomes give confidence in their use as aids to policy formulation for the use of PCT to control *A. lumbricoides* and hookworm infection. The models further largely agree in a qualitative sense on the added benefit of semi-annual *vs.* annual deworming and targeting of the entire population *vs.* only children, as well as the potential for interruption of transmission. The feasibility to implement semi-annual, population-wide PCT and achieve elimination remains subject of ongoing field studies. Further, this study also illustrates that long-term predictions are sensitive to modelling assumptions about which age groups contribute most to transmission, which in turn is a function of assumptions about human demography and age-patterns in exposure and contribution to the environmental reservoir of infection, the latter being notoriously difficult to empirically quantify.

Very different fitting techniques are used to estimate parameter values from the baseline epidemiological data. By its nature, the individual-based stochastic code requires more parameters to be estimated or derived from published work than the deterministic model. Hence arriving at initial estimates of parameters that are difficult to obtain from past published work or from the baseline data is based on a trial and error approach to iterate towards a good fit to observed epidemiological patterns (which we formalised through the use of sequential Monte Carlo (Filippi et al., 2011, Toni et al., 2009)). The deterministic model has fewer parameters and has a closed-form solution for the likelihood of observed data. However, because of the nature of the parameter combination that defines the basic reproductive number, R_0_, with reproduction and transmission parameters in the numerator and loss terms in the denominator, there is not one unique set that defines the best fit to the observed age intensity profile for either model (Supplemental Figs. S2, S3, S5, and S6). Despite both limitations, the models both predict the short-term infection levels after mass drug administration reasonably well.

So far, mathematical models for transmission and control of STH (including the two used here) assume that parasite aggregation in the host population is driven entirely by inter-individual variation in exposure to a single environmental reservoir of infection. However, it is likely that the spatial distribution of the environmental reservoir is patchy in field settings and that individuals in the population are exposed differentially to different patches. Patchiness of environmental reservoirs would promote parasite aggregation after interventions: people associated with “low intensity” patches would have lower post-control infection levels and *vice versa* for people exposed primarily to “high intensity” patches, which would result in a somewhat lower overall impact of interventions than we predict now. A complication is that patchiness of the environmental reservoir is very hard to demonstrate empirically. Still, if we were to partly attribute parasite aggregation to patchiness of the environmental reservoir, models would produce lower estimates of the level of inter-individual variation in relative exposure to the reservoir to arrive at the same effective overall level of parasite aggregation in hosts. To what extent this counterbalances the effect of patchiness would require dedicated spatial modelling studies.

Another complicating factor is that estimates of parasite aggregation can often only be informed by egg count data, which are far easier to collect than worm expulsion data. However, egg counts also suffer from high levels of variation. Ideally, more sensitive and precise DNA-based techniques (Easton et al., 2016, Llewellyn et al., 2016, Meurs et al., 2017) would be used to quantify intensity of infection reduce variation and consequent uncertainty in diagnostic test results, would be used to require mathematical models to be recalibrated to reproduce the association between worm burden and the results of such DNA-based techniques, either directly or indirectly through egg count data. The use of more precise diagnostic tests will also require the definitions for control (<1% prevalence of moderate-to-heavy intensity of infection) to be revisited, as different diagnostic tests translate a given worm burden to different levels of prevalence of infection.

What can be improved and what should be a priority for future work? First, common ground on the statistical procedures used to compare predicted outcomes with the observed pattern is desirable. Given the different implementations of the two models used here (individual-based *vs.* deterministic), it was not possible to calculate a common metric for model performance (e.g. a deviance-based statistic). In the future, this may be circumvented by using a stochastic implementation of the ICL model (Truscott et al., 2016) and/or the use of simpler metrics such as the mean root square error, although such a metric would still need to be averaged over repeated iterations of stochastic simulations. An individual-based approach will also allow more realistic estimation of prospects of elimination and will circumvent simplifying assumption required by deterministic transmission models to predict prevalence of infection (Stolk et al., 2015). Further, much information is contained within the data from individuals, so basing comparisons on the individual-level data is also desirable. To aid in this, individual-level data must be collected and stored as such, including who is treated at each round of MDA, and who gets re-infected and to what degree. On the other hand, in practice, predictions for specific situations can often only be based on limited or highly aggregated data, especially if mathematical modellers are only involved once the epidemiological data has already been collected. Therefore, it is also of interest to further compare the sensitivity of the predictive performance of different models for the level of data aggregation and the additional modelling assumptions that then need to be made. Further, broadening the model comparisons to prospects of interruption of transmission and inclusion of other STH species such as *Trichuris trichiura* are important next steps for the collaborative work between the two modelling teams. Again, good baseline and follow-up data sets post treatment for *T. trichiura* are required to facilitate these comparisons of predictive ability. Last, for both stochastic and deterministic models, clear definitions and quantification of the correlations between parameters are essential (Supplemental Figs. S2, S3, S5, and S6), and need to be carried forward in predictions for prospects of control and elimination of STH.

Overall, however, the good fits to the detail of the observed epidemiological patterns described here are very encouraging. In part, this is a consequence of the predictable dynamics of helminth parasites within human communities where, as noted in the introduction, post treatment, the parasite infection levels bounce back in a monotonic manner to their pre-treatment equilibrium, in the absence of other changes in the environment.

## Competing interests

RMA is a non-executive director of the board of GlaxoSmithKline (GSK). GSK had no influence on the conduct of the research, its funding, or the writing of this paper.

All other authors declare no competing interests.

## Authors’ contributions

Conception and design of the study: SJdV and RMA. Acquisition of data: RS and GK. Analysis and interpretation of data: LEC, JET, SHF, and HCT. Drafting the article: LEC and JET. Critical revision of manuscript for important intellectual content: SJdV, RMA, RS, GK, LEC, JET, SHF, and HCT. All authors approved the final article submitted.

## Figures and Tables

**Table 1:** Overview and comparison of model structures and implementation. Supplemental Table S1 provides a detailed overview of the parameterisation and quantification of both models with detailed references to sources.

Model structure	ICL model	Erasmus MC model
Simulation framework	Deterministic	Stochastic individual-based (humans and worms) and deterministic (environmental reservoir of infection)

Implementation language	R	Java

Inter-individual variation in exposure and contribution to the reservoir	By age (piece-wise constant) and random individual factors (governed by parasite aggregation parameter)	By age (piece-wise linear) and random individual factors (governed by parasite aggregation parameter)
* Hookworm*	Age-specific exposure and contribution are assumed to be proportional	Age-specific exposure and contribution are assumed to be proportional (both largely depend on skin contact to the environmental reservoir)
* Ascariasis*	Age-specific exposure and contribution are assumed to be proportional	Age-specific exposure peaks in children and declines thereafter (because it depends on ingestion of contaminated soil), but contribution rises with age and is stable from age ten onwards (because it depends on defaecation practices) as previously estimated for hookworm

Inter-individual variation in host suitability for female worms to produce eggs in	None assumed	Assumed to follow a gamma distribution

Acquired immunity to infection	No acquired immunity assumed	No acquired immunity assumed

Coverage of and compliance to preventive chematherapy	Structured by age group	Probability of participation varies with age and between individuals due to random personal factors

Reproductive model	Mating function for dioecious polygamous parasite	Explicit simulation of prepatent period and insemination of female worms by polygamous male worms
