# Supplementary material for: Comparison and validation of two mathematical models for the impact of mass drug administration on Ascaris lumbricoides and hookworm infection
Source: Epidemics. 2017 Mar;18:38–47. doi: 10.1016/j.epidem.2017.02.001 (PMC5340859; doi:10.1016/j.epidem.2017.02.001)
Supplement: Supplementary file 1 [file mmc1.docx]

## Supplemental File legend

**Supplemental Table 1:** Model parameters used to simulate transmission of *Ascaris lumbricoides* and hookworm infections.

**Supplemental Figure 1:** Schematic representation of the hookworm field trial timeline.

**Supplemental Figure 2:** Correlation plot of posterior samples of parameter values for the Erasmus MC model for ascariasis. Open circles represent sampled parameter values where the diameter of each circle is proportional to the square root of the posterior mass it represents.

**Supplemental Figure 3:** Marginal scatterplots of sampled values from the posterior distribution of fitted parameters for the ICL model for Ascaris. The beta value (age-dependent relative exposure and contribution to the reservoir) for the 10– 20 age group is set to 1.

**Supplemental Figure 4:** Comparison of parameter estimates for age-dependent exposure to the environmental reservoir of infection for transmission of *A. lumbricoides*. Estimated age-dependent exposure generated predicted levels of infection as illustrated in Figure 1 (left panel). The scales of relative exposure differ between the two models because of different choices of reference age for relative exposure of 1.0, however, this has no repercussions for model predictions as the total area under each curve scales with other transmission parameters in both models.

**Supplemental Figure 5:** Correlation plot of posterior samples of parameter values for the Erasmus MC model for hookworm. Open circles represent sampled parameter values where the diameter of each circle is proportional to the square root of the posterior mass it represents.

**Supplemental Figure 6:** Marginal scatterplots of sampled values from the posterior distribution of fitted parameters for the ICL model for hookworm. The beta value (age-dependent relative exposure and contribution to the reservoir) for the 15 – 25 age group is set to 1.
