# Supplementary material for: Comparison and validation of two mathematical models for the impact of mass drug administration on Ascaris lumbricoides and hookworm infection
Source: Epidemics. 2017 Mar;18:38–47. doi: 10.1016/j.epidem.2017.02.001 (PMC5340859; doi:10.1016/j.epidem.2017.02.001)
Supplement: Supplementary file 2 [file mmc2.docx]

Supplemental Table 1. Model parameters used to simulate transmission of *Ascaris lumbricoides* and hookworm infections.

|  | **Value or assumption** | |
| --- | --- | --- |
| **Parameter** | **Erasmus MC** | **Imperial College London** |
| **Human demography** | | |
| *Hookworm* | Indian demography previously used for simulating transmission and control of lymphatic filariasis through to 2008 [1]. | Demographic data taken from a detailed STH study in Uganda – the pattern is very similar to that pertaining in India at present [2]. |
| *Ascariasis* | Indian fertility and mortality rates as reported for 1980-1985 by United Nations Population Division (2015 Revision). | Demographic data taken from a detailed STH study in Uganda - the pattern is very similar to that pertaining in India at present [2]. |
| **Transmission of infection** | | |
| Seasonal variation in contribution to reservoir | Stable throughout the year (assumption). | Stable throughout the year (assumption). |
| Aggregation of parasites in hosts |  |  |
| *Hookworm* | $k_{w}=0.24$, estimated from pre-control epidemiological data from Tamil Nadu [3], conditional on estimate of measurement error in repeated McMaster egg counts (see part of this table on diagnostic test outcomes). | $k_{w}=0.35$, estimated from pre-control epidemiological data from Tamil Nadu [3], conditional on estimate of measurement error in repeated McMaster egg counts (see part of this table on diagnostic test outcomes). |
| *Ascariasis* | $k_{w}=0.8$ [4]. | $k_{w}=0.9$ [4,5]. |
| Variation in exposure and contribution to the environmental reservoir by age and sex |  |  |
| *Hookworm* | Relative exposure and contribution to the reservoir both increase linearly from 0 to 1 between ages 0–10 and is stable thereafter with no difference between males and females [6]. | Relative exposure and contribution to the reservoir are assumed to vary piece-wise constant by age group, and are estimated at 0.12 (ages 0-15), 1 (ages 15-25), and 0.07 (ages 25+), assuming no difference between males and females. These figures were estimated from unpublished epidemiological data from Tamil Nadu. |
| *Ascariasis* | Contribution to the reservoir increases linearly from 0 to 1 between ages 0–10 and is stable thereafter with no difference between males and females (reflecting behaviour related to defaecation and mobility patterns as previously estimated for hookworm [6]). Exposure to the reservoir is defined as a piece-wise linear function of age that increases linearly from a base level $x_{0}$ of relative exposure at age zero to a relative exposure of 1.0 at age $a_{\text{peak}}$, and then again linearly declines back to the base level $x_{0}$ at age 20, and is stable thereafter. This function aims to reflect behaviour leading to ingestion of contaminated matter, which typically peaks in young children. The base level of relative exposure $x_{0}$ (0.33 ±0.041 SD) and peak age $a_{\text{peak}}$ (2.9 ±0.77 SD) were estimated from the data on pre-control age patterns in average worm burden [4]. | Relative exposure and contribution to the reservoir by age are assumed to be equal, and are estimated from the baseline data: 1.6 (0-4 years), 1.54 (5-9), 1.0 (10-19), 0.9 (20-29), 0.6 (30-40) and 0.5 (45-70) [age categories chosen to match data]. |
| **Life history and productivity of the parasite in the human host** | | |
| Average worm lifespan |  |  |
| *Hookworm* | 3 years [7–9]. | 2 years [10]. |
| *Ascariasis* | 1 year [4,7–9,11]. | 1 year [7–9,11]. |
| Variation in worm lifespan | Weibull distribution with shape 2; i.e. the mortality rate is zero at age zero and then increases linearly with worm age (assumption as previously used for hookworm [6]). | Exponential distribution; i.e. the mortality rate is constant and independent of worm age. |
| Pre-patent period |  |  |
| *Hookworm* | 7 weeks [7,8,12,13]. | No pre-patent period used. |
| *Ascariasis* | 10 weeks [7]. | No pre-patent period used. |
| Age-dependent reproductive capacity | Constant over age (assumption). | Constant over age (assumption). |
| Female worm fecundity | Density-dependent on total number of female worms in host, assuming hyperbolic saturation [6]. | Density-dependent on total number of female worms in host, assuming exponential saturation. Exponential model of saturation with parameter γ = 0.02 for hookworm (fitted to data) and γ = 0.07 for hookworm [5]. |
| *Hookworm* | On average 4 eggs per female worm per 50 mg sample of faeces (200 epg per female worm, as previously reported based on association between number of expulsed adult female worms and egg counts based on Kato-Katz [14]). The average maximum total host output is assumed to be 30 eggs per 50 mg faeces (1500 epg, as previously assumed [6]). | On average 4 eggs per female worm per 50 mg sample of faeces (200 epg per female worm, as previously reported based on association between number of expulsed adult female worms and egg counts based on Kato-Katz [14]). |
| *Ascariasis* | On average 195 eggs per female worm per 50 mg sample of faeces (9729 epg per female worm), and maximum total host output of 373 eggs per 50 mg faeces on average (18,675 epg). These figures were estimated from pre-control data on number of expulsed adult female worms and egg counts based on a concentration and sedimentation technique using homogenised stools [4]. | Ascaris validation carried out using expelled worm data, so egg production not required. |
| Variation in worm fecundity due to host suitability | Defined as inter-individual variation in relative maximum total host egg output, as described by a gamma distribution with mean 1.0 and a value of 50 for both the shape and rate parameters (95%-CI: 0.74–1.30) [6]. | None (assumption). |
| Host immunity to incoming infections | None (assumption). | None (assumption). |
| **Infection dynamics in environmental reservoir** | | |
| Survival of infective material in the central reservoir | Exponential survival (assumption). | Exponential survival (assumption). |
| *Hookworm* | Average lifespan of two weeks, implemented as a monthly survival probability of $\exp\left( -26/12 \right)=11.5\%$ (95%-CI: 0.05–7.38 weeks under assumption of exponential survival), based on the notion that average survival time is in the order of weeks [12,13,15]. | Average lifespan of 12 days [10]. |
| *Ascariasis* | Average lifespan of 1.5 month, implemented as a monthly survival probability of $\exp\left( -1/1.5 \right)=51.3\%$s (95%-CI: 0.04–5.53 months under assumption of exponential survival) [8,9]. | Lifespan of approximately 2 months [16]. |
| **Drug treatment** | | |
| Proportion of adult worms killed by single dose of albendazole (400 mg), or pyrantel pamoate (10 mg/kg, ascariasis only) | Assumption: proportion killed is equal to the faecal egg reduction rate. | Assumption: proportion killed is equal to the faecal egg reduction rate. |
| *Hookworm* | 0.962 for albendazole [17]. | 0.940 for albendazole [18]. |
| *Ascariasis* | 0.999 for albendazole [17], and 0.95 for pyrantel pamoate [4]. | 0.990 for albendazole [18], and 0.95 for pyrantel pamoate [4]. |
| **Diagnostic test outcomes** |  |  |
| Variability in measured host load of infective material (eggs per examined sample of faeces) |  |  |
| *Hookworm* | McMaster: negative binomial distribution with aggregation parameter $k=0.19$ (estimated separately outside the transmission model), estimated from triple egg count data from, using only data from individuals with at least one count above 0 (estimated from unpublished data from Tamil Nadu, India, which were also used to validate the model). | McMaster: negative binomial distribution with aggregation parameter $k=0.33$ (estimated separately outside the transmission model), estimated from triple egg count data from (unpublished data from Tamil Nadu, India, which were also used to validate the model). |
| *Ascariasis* | Not applicable for model validation, which was based on worm counts. | Not applicable for model validation, which was based on worm counts. |
| Cut-offs for no, light, moderate, and heavy infection |  |  |
| *Hookworm* | 1, 2000, and 4000 epg [19]. | 1, 2000, and 4000 epg [19]. |
| *Ascariasis* | 1, 1000, and 10,000 epg [19]. | 1, 1000, and 10,000 epg [19]. |

## References

1. Jambulingam P, Subramanian S, de Vlas SJ, Vinubala C, Stolk WA (2016) Mathematical modelling of lymphatic filariasis elimination programmes in India: required duration of mass drug administration and post-treatment level of infection indicators. *Parasit Vectors* **9**: 501.

2. Pullan RL, Kabatereine NB, Quinnell RJ, Brooker S (2010) Spatial and Genetic Epidemiology of Hookworm in a Rural Community in Uganda. *PLoS Negl Trop Dis* **4**: e713.

3. Sarkar R, Rose A, Mohan VR, Ajjampur SSR, Veluswamy V, et al. (2017) Study design and baseline results of an open-label cluster randomized community-intervention trial to assess the effectiveness of a modified mass deworming program in reducing hookworm infection in a tribal population in southern India. *Contemp Clin Trial Comm* **5**: 49–55.

4. Elkins DB, Haswell-Elkins M, Anderson RM (1986) The epidemiology and control of intestinal helminths in the Pulicat Lake region of Southern India. I. Study design and pre- and post-treatment observations on Ascaris lumbricoides infection. *Trans R Soc Trop Med Hyg* **80**: 774–792.

5. Truscott JE, Turner HC, Farrell SH, Anderson RM (2016) Soil-Transmitted Helminths: Mathematical Models of Transmission, the Impact of Mass Drug Administration and Transmission Elimination Criteria. *Adv Parasitol* **94**: 133–198.

6. Coffeng LE, Bakker R, Montresor A, de Vlas SJ (2015) Feasibility of controlling hookworm infection through preventive chemotherapy: a simulation study using the individual-based WORMSIM modelling framework. *Parasit Vectors* **8**: 541.

7. Bethony J, Brooker S, Albonico M, Geiger SM, Loukas A, et al. (2006) Soil-transmitted helminth infections: ascariasis, trichuriasis, and hookworm. *Lancet* **367**: 1521–1532.

8. Anderson RM, Truscott J, Hollingsworth TD (2014) The coverage and frequency of mass drug administration required to eliminate persistent transmission of soil-transmitted helminths. *Philos Trans R Soc L B Biol Sci* **369**: 20130435.

9. Truscott JE, Hollingsworth TD, Brooker SJ, Anderson RM (2014) Can chemotherapy alone eliminate the transmission of soil transmitted helminths? *Parasit Vectors* **7**: 266.

10. Anderson RM, May RM (1985) Helminth infections of humans: mathematical models, population dynamics, and control. *Adv Parasitol* **24**: 1–101.

11. Croll NA, Anderson RM, Gyorkos TW, Ghadirian E (1982) The population biology and control of Ascaris lumbricoides in a rural community in Iran. *Trans R Soc Trop Med Hyg* **76**: 187–197.

12. Hotez PJ, Brooker S, Bethony JM, Bottazzi ME, Loukas A, et al. (2004) Hookworm infection. *N Engl J Med* **351**: 799–807.

13. Brooker S, Bethony J, Hotez PJ (2004) Human Hookworm Infection in the 21st Century. *Adv Parasitol*. Vol. 58. pp. 197–288.

14. Anderson RM, Schad GA (1985) Hookworm burdens and faecal egg counts: an analysis of the biological basis of variation. *Trans R Soc Trop Med Hyg* **79**: 812–825.

15. Augustine DL (1923) Investigations on the control of hookworm disease. XVI. Length of life of hookworm larvae from the stools of different individuals. *Am J Epidemiol* **3**: 127–136.

16. Anderson RM, May RM (1982) Population dynamics of human helminth infections: control by chemotherapy. *Nature* **297**: 557–563.

17. Levecke B, Montresor A, Albonico M, Ame SM, Behnke JM, et al. (2014) Assessment of anthelmintic efficacy of mebendazole in school children in six countries where soil-transmitted helminths are endemic. *PLoS Negl Trop Dis* **8**: e3204.

18. Vercruysse J, Behnke JM, Albonico M, Ame SM, Angebault C, et al. (2011) Assessment of the anthelmintic efficacy of albendazole in school children in seven countries where soil-transmitted helminths are endemic. *PLoS Negl Trop Dis* **5**: e948.

19. Albonico M, Bickle Q, Ramsan M, Montresor A, Savioli L, et al. (2003) Efficacy of mebendazole and levamisole alone or in combination against intestinal nematode infections after repeated targeted mebendazole treatment in Zanzibar. *Bull World Heal Organ* **81**: 343–352.
