# Supplementary figures and images for: Comparison and validation of two mathematical models for the impact of mass drug administration on Ascaris lumbricoides and hookworm infection
Source: Epidemics. 2017 Mar;18:38–47. doi: 10.1016/j.epidem.2017.02.001 (PMC5340859; doi:10.1016/j.epidem.2017.02.001)

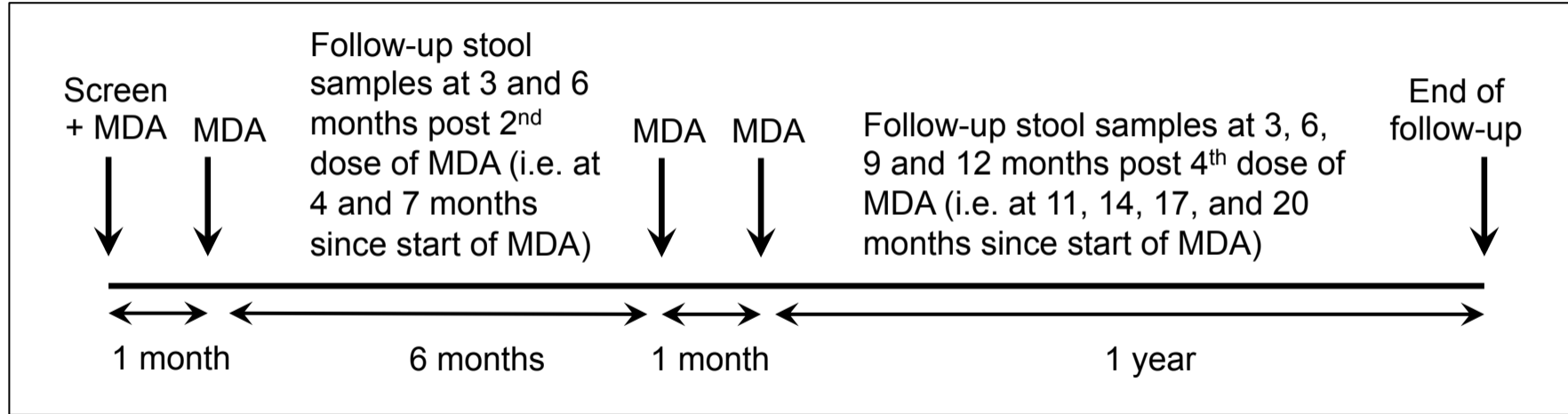

Supplement: Supplementary file 3 [file mmc3.pdf]

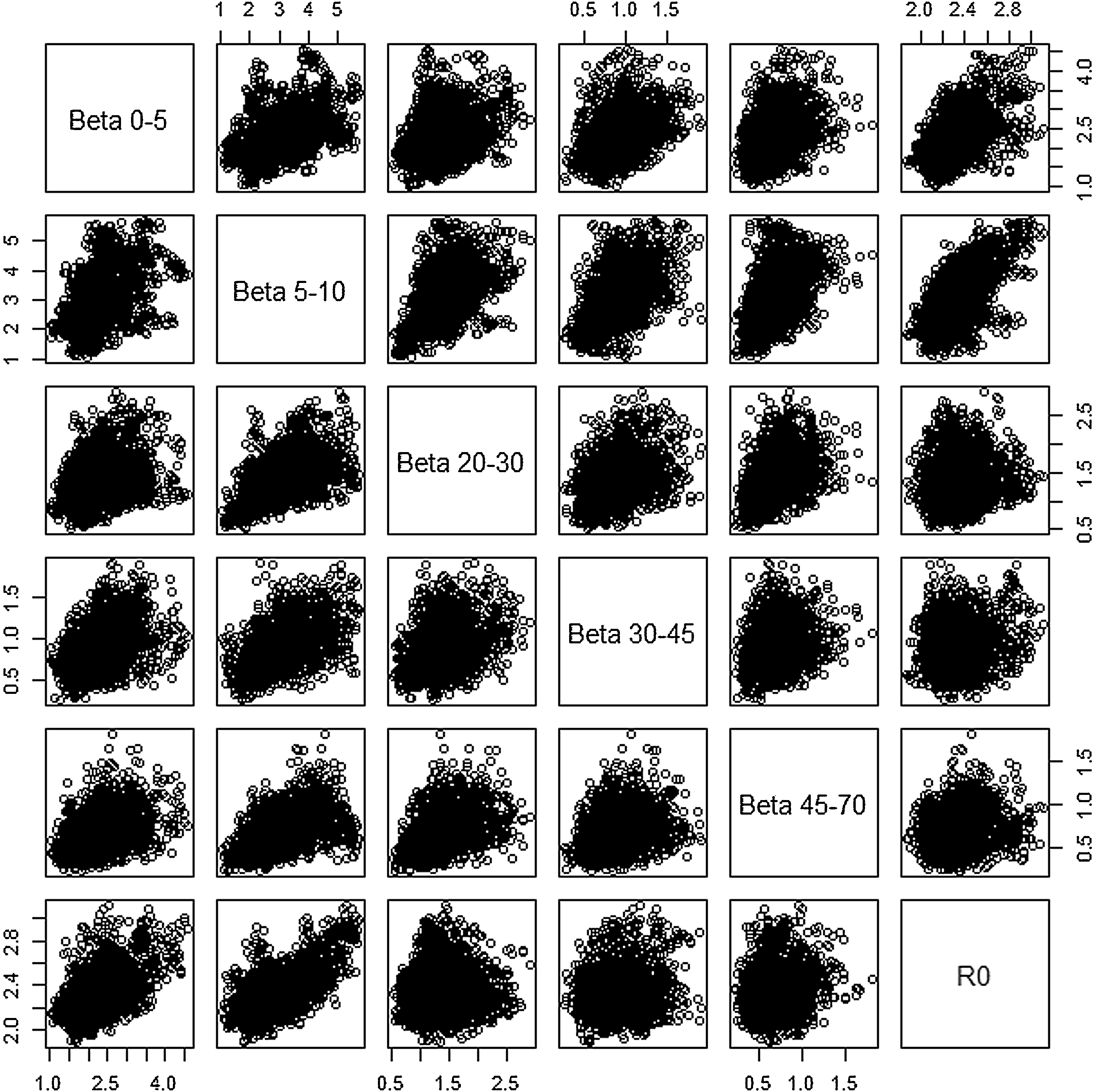

Supplement: Supplementary file 4 [file mmc4.jpg]

# Ascaris correlation plot of posterior parameter values

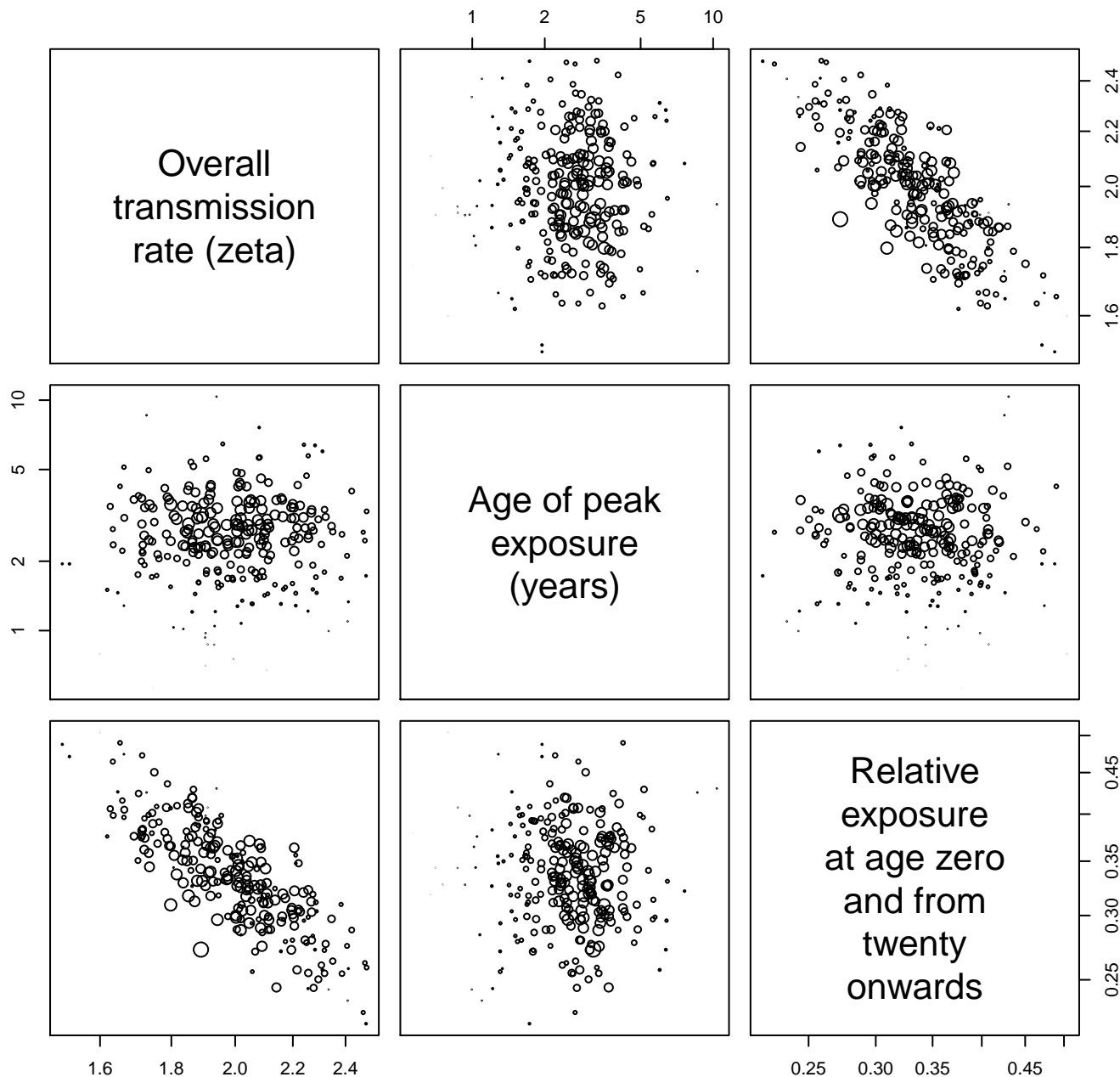

Supplement: Supplementary file 5 [file mmc5.pdf]

## Ascaris: comparison of estimates for relative exposure to the reservoir by age

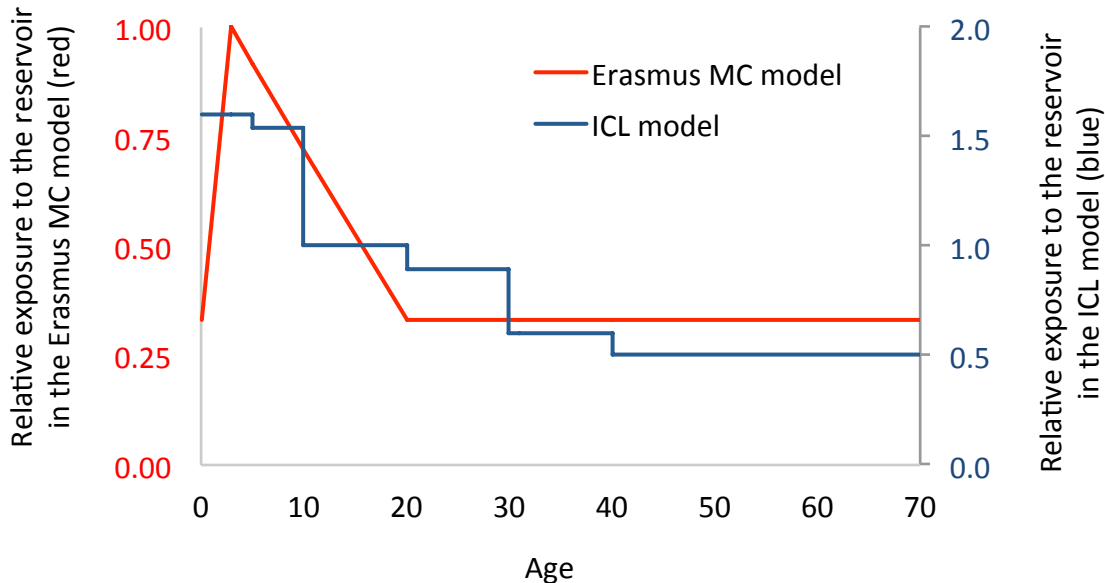

Supplement: Supplementary file 6 [file mmc6.pdf]

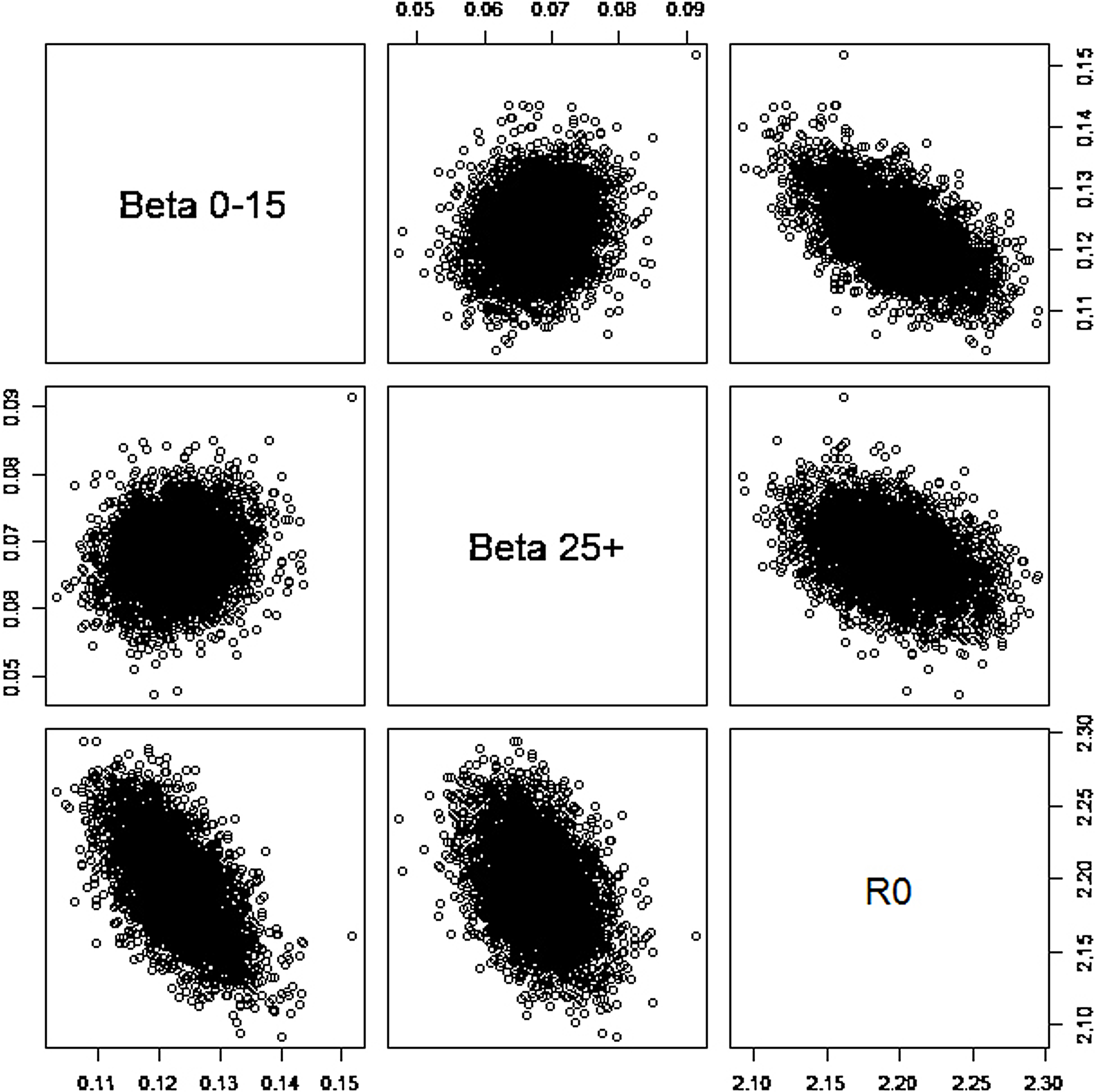

Supplement: Supplementary file 7 [file mmc7.jpg]

**Hook worm correlation plot of posterior parameter values**

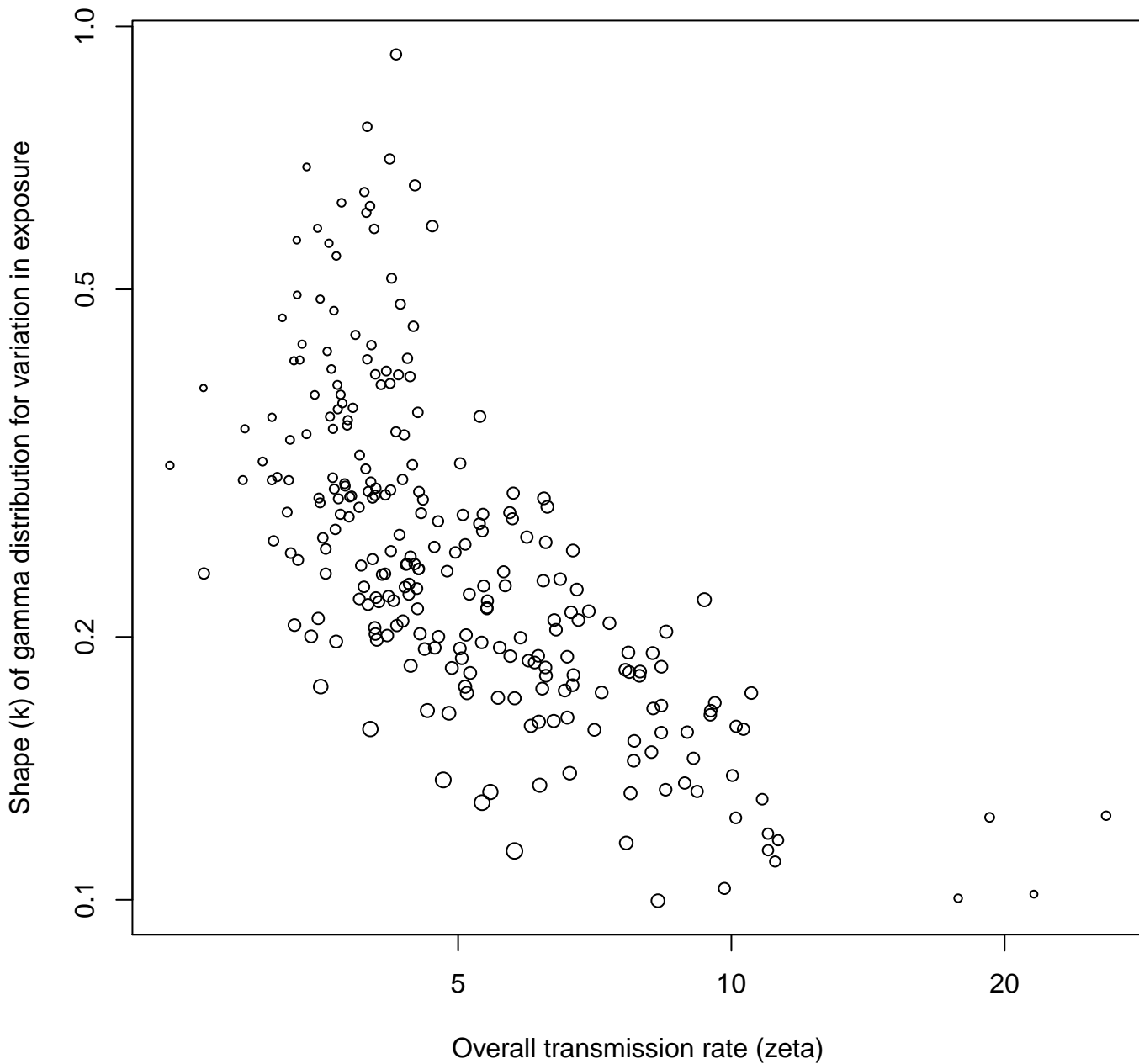

Supplement: Supplementary file 8 [file mmc8.pdf]
